# Combined semiquantitative nail-enthesis complex ultrasonography and capillaroscopy in psoriasis and psoriatic arthritis

**DOI:** 10.3389/fimmu.2024.1505322

**Published:** 2025-01-14

**Authors:** Giacomo Cafaro, Roberto Bursi, Valentina Valentini, Katharina Hansel, Carlo Perricone, Vincenzo Venerito, Onelia Bistoni, Manuela Sebastiano, Fabiana Topini, Luca Stingeni, Roberto Gerli, Elena Bartoloni

**Affiliations:** ^1^ Rheumatology Unit, Department of Medicine and Surgery, University of Perugia, Perugia, Italy; ^2^ Dermatology Section, Department of Medicine and Surgery, University of Perugia, Perugia, Italy; ^3^ Rheumatology Unit, Department of Precision and Regenerative Medicine and Ionian Area (DiMePRe-J), University of Bari Aldo Moro, Bari, Italy

**Keywords:** psoriasis, psoriatic arthritis, rheumatoid arthritis, ultrasound, capillaroscopy

## Abstract

This pilot study investigates distinctive features within the nail-enthesis complex among Psoriatic arthritis (PsA), Psoriasis (PSO), Rheumatoid Arthrit is (RA), and Healthy Control (HC) groups, utilizing a combined approach of ultrasound (US) and nailfold videocapillaroscopy (NVC). Clinical assessments and comprehensive US and NVC evaluations of the nail-enthesis complex were conducted on 72 subjects (18 PsA, 16 PSO, 19 RA, 19 HC). Unsupervised clustering models and factor analysis were employed to identify patterns and interrelationships between US and NVC parameters. Significant structural differences were detected, emphasizing the discriminatory power of semiquantitative US scores (GS BUNES, Wortsman type). Trends in vascularization aligned with literature, showcasing dysregulated angiogenesis in PsA and PSO. The clustering model effectively distinguished HC from PsA subjects, revealing a potential continuum between PSO and PsA. RA subjects exhibited subsets with features akin to both HC and PsA/PSO, underscoring the complexity of its manifestations. This study provides insights into nail-enthesis complex alterations, highlighting distinctions among PsA, PSO, RA, and HC subjects. The clustering model emphasizes potential overlap between PSO and PsA. Factor analysis elucidates collinearity in US-detected characteristics, while suggesting limited discriminative power of some quantitative parameters. These findings advocate for further exploration in prospective trials, potentially predicting the evolution of undifferentiated early arthritis and arthritis onset in PSO patients.

## 1 Introduction

Psoriatic arthritis (PsA), skin psoriasis (PSO), and rheumatoid arthritis (RA) are distinct inflammatory conditions characterized by their unique clinical features and disease presentations. PsA, affecting up to 40% of individuals with PSO, displays a heterogeneous spectrum of manifestations, including enthesitis, dactylitis, and axial involvement, alongside peripheral arthritis. PSO primarily manifests as a chronic dermatitis, typified by characteristic cutaneous plaques, while RA predominantly involves symmetrical polyarthritis, synovitis, and the potential for systemic complications (1, 2).

The nail-enthesis complex, an essential junction between tendons, ligaments, and adjacent bone structures, represents a critical site in the pathogenesis of various arthritic conditions. Comprising the intricate connections between the nail unit, the distal interphalangeal joint, and surrounding soft tissues, this complex plays a pivotal role in maintaining digit functionality (3, 4). Utilizing ultrasound (US) imaging for the assessment of the nail-enthesis complex has proven valuable, enabling the visualization of subtle structural and vascular changes that mirror underlying pathological processes (5). These changes include alterations in nail plate, bed and matrix, periungual tissue abnormalities, and variations in the adjacent bone interface (3).

The observed vascular changes within the nail-enthesis complex reflect the dysregulated angiogenesis observed in both PSO and PsA, highlighting shared pathogenic underpinnings between psoriasis skin lesions and the joint manifestations observed in PsA (6). Specifically, these alterations manifest as abnormal vascular morphology and dysregulated angiogenic growth factors, distinguishing them from the distinct angiogenic profiles identified in rheumatoid arthritis (6). Moreover, neoangiogenesis changes described in the PsA synovium during arthroscopy reveal unique patterns, differing from those observed in RA, further emphasizing the distinct pathogenic mechanisms at play in these conditions (6).

Nailfold videocapillaroscopy (NVC), emerging as a valuable tool for assessing microvascular changes in various inflammatory arthritides, allows for the direct visualization of capillary morphology and architecture (7, 8). It is an economical, repeatable, and easily accessible methods that can aid in the identification of specific microvascular patterns associated with psoriasis and different arthritic conditions, including PsA, RA, and early arthritis (7, 9). While existing literature has demonstrated promising results, discrepancies in findings underline the need for further investigations to establish a comprehensive and reliable diagnostic framework (10).

This pilot study aims to explore the potential of a combined approach utilizing nail-enthesis complex US and NVC (11). By harnessing the synergistic capabilities of these two modalities, this research seeks to enhance the accuracy and precision in differentiating between patients with PsA, PSO and RA, distinguishing them from healthy individuals (12) through the identification of a subset of US and capillaroscopic parameters that can be effectively employed in routine clinical settings. To further identify variables capable of differentiating among the conditions, considering the limited number of subjects included in a pilot study, an unsupervised clustering model exclusively based on US and NVC parameters was employed.

## 2 Patients and methods

### 2.1 Patients, controls and clinical assessment

Consecutive enrolment of twenty patients with PsA fulfilling CASPAR classification criteria (13) was conducted at the outpatient clinic. Twenty age- and sex-matched controls for each RA (according to 2010 ACR/EULAR criteria (14)), PSO and healthy control (HC) groups were selected. Exclusion criteria included subjects with concomitant systemic autoimmune diseases, acrocyanosis and Raynaud’s phenomenon. Disease activity, ongoing treatment (topical, immunosuppressors, glucocorticoids, etc.), and the type of involvement (nail psoriasis, inverse psoriasis, enthesitis, arthritis, dactylitis, axial involvement, etc.) were not specific exclusion criteria. A comprehensive clinical assessment was performed by a blinded team consisting of a rheumatologist and a dermatologist who were not aware of patient’s diagnosis and of the results of each other’s assessment. Blinding was obviously not applicable in case of overt skin psoriasis, though the investigators were not aware whether the patients had musculoskeletal involvement or not. Essential parameters, such as age, sex, body mass index (BMI), swollen joint count on 66 joints, tender joint count on 68 joints, Disease Activity Score on 28 joints (DAS28)-CRP, Disease Activity Index for Psoriatic Arthritis (DAPSA), Maastricht Ankylosing Spondylitis Enthesitis Score (MASES), Psoriasis Area Severity Index (PASI), and Nail Psoriasis Severity Index (NAPSI), were evaluated.

Additional recorded parameters included C-reactive protein (CRP), erythrocyte sedimentation rate (ESR), rheumatoid factor (RF), anti-citrullinated peptide antibodies (ACPA), and ongoing treatment with conventional synthetic disease-modifying antirheumatic drugs (csDMARD) or biologic/target synthetic DMARD (b/tsDMARD).

The study was approved by the local ethics committee *Comitato Etico Regionale Umbria* (23981/22/ESS - 23/02/2022) and all patients provided informed consent to participation.

### 2.2 Ultrasound assessment

Each participant underwent a comprehensive US assessment of the nail-enthesis complex. This evaluation entailed a thorough examination of the nail plate, matrix bed, and the extensor digitorum tendon enthesis through longitudinal scans. The assessment was performed by experienced rheumatologists with expertise in musculoskeletal and soft tissue ultrasonography. Measures were taken to minimize bias, with the operator conducting the US blinded to the clinical information. Since blinding was not possible for patients with evident skin psoriasis and nail involvement, scoring was performed on anonymized and randomized recorded images, minimizing potential bias. An ESAOTE MyLabSeven machine (ESAOTE, Genoa, Italy) equipped with a 19 MHz linear probe was utilized for the US assessment. GS gain was set between 60% and 80% as appropriate, pulse repetition frequency was set at 750 Hz, PD gain was set just below the threshold at which background artifacts appeared.

**Figure 1:**
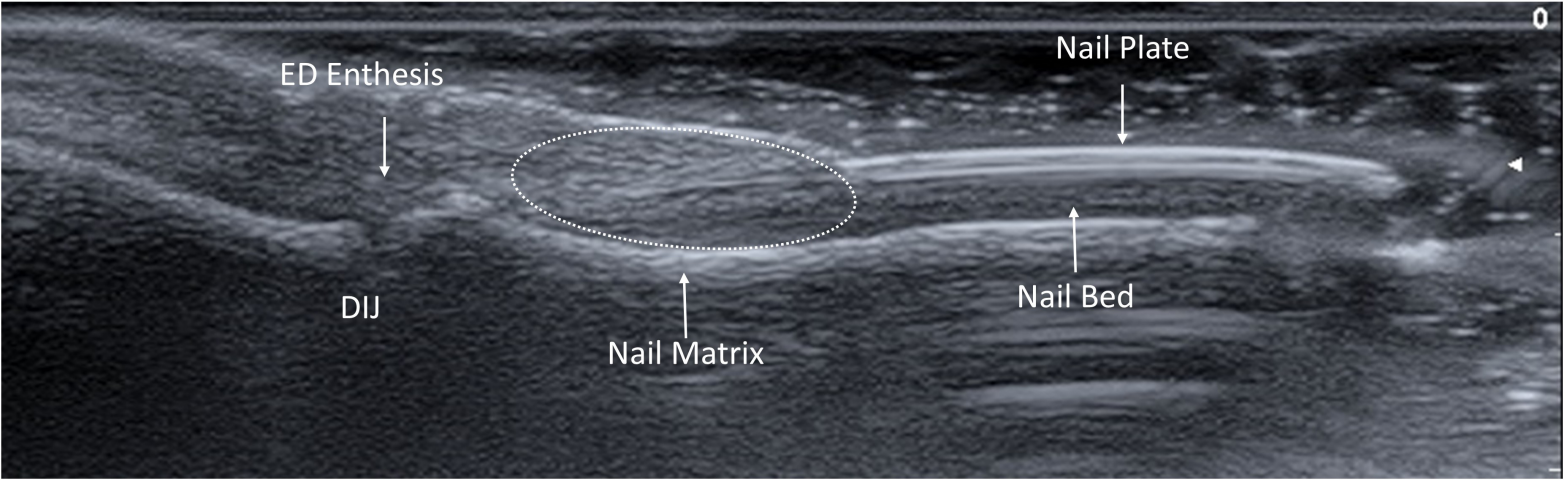
Ultrasound anatomy of the nail-enthesis complex. The nail-enthesis complex is made of multiple structures. The extensor digitorum ED tendon merges with the DIJ capsule and further branches into two components. The deep fibers attach to the bone cortex of the distal phalanx, while the superficial fibers represent the supporting scaffold of the nail matrix, from which the nail plate develops and extends distally to for a trilaminar structure with two linear hyperechoic bands split by a hypoechoic one. The nail plate is held in place by the nail bed that connects it with the bone cortex of the distal phalanx. ED, Extensor Digitorum; DIJ, Distal Interphalangeal Joint.

Specific parameters including the Wortsman type (15), enthesitis score and the semiquantitative GS BUNES and PD BUNES scores were assessed according to defined criteria (10). The scoring methodology entailed careful consideration of various factors, including the nail matrix, bed, and plate. Additionally, measurements were taken for nail plate thickness, bed thickness, and matrix thickness, with each structure measured independently three times to ensure accurate calculations. Nail plate thickness was measured from the surface of the dorsal plate to the bottom of the ventral plate. Bed thickness was measured from the bottom of the ventral plate to the bone cortex of the distal phalanx. Matrix thickness was measured from the bone cortex, perpendicularly to the dermal-hypodermal interface (**Figure 1**). Extensor digitorum enthesis was assessed in GS and PD, providing a score of 0-3 and 0-1 for each of the parameters, according to OMERACT definition (16).

For each score, the mean value of the ten digits was computed. Fingernails with previous significant trauma, surgery or evidence of infection were excluded. To avoid bias caused by the effect of room temperature on vasodilation of the extremities, all patients, before undergoing US and NVC, were kept in a room with controlled temperature between 20 and 23°C for at least 20 minutes.

### 2.3 Nailfold videocapillaroscopy

NVC was conducted using the VideoCap^®^ 3.0 workstation (DS Medica, Milan, Italy) by an experienced rheumatologist (VV). The examiner was blinded to the clinical and US data to avoid potential biases during the assessment. NVC assessment was performed according to the consensus of the EULAR Study Group on Microcirculation in Rheumatic Diseases (17). Briefly, the evaluation involved an examination of digits from the 2nd to 5th fingers in both hands. Recorded parameters included capillary density (number of capillaries per mm as the mean of at least three independent fields), the presence of hemosiderin deposits, the number of tortuous (≥ 2 crossings between afferent and efferent limbs) and dilated capillaries (apical diameter ≥ 20 µm), as well as giant capillaries and ramified capillaries (at least 3 branches originating from a single normal-sized capillary). Mean values of the 8 digits were calculated and used for data analysis. In case of very low visibility due to edema, to avoid missing significant changes, NVC findings of single digits were excluded from the analysis.

### 2.4 Data analysis

The variables collected are shown as mean ± standard deviation (SD) or as absolute number (percentage), as appropriate. Comparisons of continuous variables among multiple groups were evaluated by Kruskal-Wallis test, followed by pairwise analysis with Bonferroni correction. Comparison of binary variables among group was assessed by χ^2^ test. Inter-rater reliability was assessed by Cohen’s kappa (18).

After variable scaling, two unsupervised clustering models were built (i.e. a k-means clustering model and a deep embedded clustering model – DEC) only including US and NVC data, along with a variable identifying the group each subject belonged to (HC, PSO, PsA or RA). The number of clusters was established with the elbow method. K-means clustering models are a mainstay of unsupervised machine learning. However, more recently a DEC method was developed (19). It combines Autoencoder with K-means for clustering rather than dimensionality reduction. The Workflow for DEC required 10 steps:

- Step 1: Estimating the number of clusters
- Step 2: Creating and training a K-means model
- Step 3: Creating and training an autoencoder
- Step 4: Implementing DEC Soft Labeling
- Step 5: Creating a new DEC model
- Step 6: Training the New DEC Model
- Step 7: Using the Trained DEC Model for Predicting Clustering Classes
- Step 8: Jointly Refining DEC Model
- Step 9: Using Refined DEC Model for Predicting Clustering Classes
- Step 10: Comparing with K-means

Accurate pre-processing with feature scaling and autocorrelation checks was carried out. Observations with missing data were ablated. In Steps 1-2, the k-means algorithm was used, minimizing the sum of the squared variance per cluster from the cluster center. In steps 3-5, a DEC model was implemented using an auto-encoder for dimensionality reduction and a clustering layer for cluster identification. This implies the stepwise unsupervised reduction of input variables to a set of representative essential features. The model training was conducted in steps 6-7: in a layer-wise pre-training and across-layer fine-tuning of the auto-encoder, the model weights were initialized. Then, to initialize the cluster centers (centroids) for the final clustering training, the data were passed through the initialized deconvolutional neural network, and standard k-means clustering was performed on the embedded data points. The clustering was then refined by training the DEC model, optimizing the Kullback-Leibler divergence in step 8. All training steps were performed using the same independent train partition of the data. In step 9, the validation of the clustering algorithm was performed using 10 random initializations and comparing the yielded clusters by assignment overlap and clinical meaningfulness. To assess the clustering performance, a t-distributed stochastic neighbor embedding (t-SNE) plot (20) was created and compared with K-means in step 10.

The most performing model was selected for subsequent analysis, in which the model was run including exclusively the US and NVC data, thus removing the variable identifying the groups.

In order to further understand how the variables influenced the distribution of the subjects among the clusters, the dataset was then tested with Bartlett’s test of sphericity and Keiser-Meyer-Olkin (KMO) and factor analysis carried out; factors with eigenvalues ≥ 1 were selected and loadings calculated. Factor analysis is a dimensionality reduction technique that allows to extract the variance shared among multiple variables, reducing them into a smaller set of factors.

Clusters were compared according to the variables collected as described above. Differences were considered significant for p<0.05.

The analysis was performed in a Python 3.9 environment using Numpy 1.23.4, Scipy 1.9.3, Scikit-learn 1.1.3, Keras API 2.10, Matplotlib 3.5.3, Seaborn 0.12.1, Pandas 1.5.1, and XGBoost 1.5.1.

## 3 Results

The initial cohort included a total of 80 subjects of whom 72 were finally analyzed. One PSO patient was excluded for a previously undiagnosed Raynaud’s phenomenon while 1 subject from the HC group, 1 from the RA group, 2 from the PsA group, and 3 from the PSO group had missing values. Patient demographic and clinical characteristics are illustrated in detail in **Table 1**. The study cohorts displayed overall comparability. As expected, PsA and RA groups were characterized by significant increased number of tender and swollen joint counts in comparison to other groups. Moreover, a higher proportion of RA patients were undergoing treatment with csDMARDs.

**Table 1:** Demographic and disease-related characteristics of the study cohort.

	HC N=19	RA N=19	PsA N=18	PSO N=16	p
Age	58 ± 12	57 ± 12	58 ± 11	56 ± 11	0.901
Disease duration	n/a	8.1 ± 6.0	7.7 ± 8.4	16.7 ± 16.5	0.348
Sex (M)	9/19 (47.4)	9/19 (47.4)	10/18 (55.6)	7/16 (43.8)	0.914
Height	170 ± 8	171 ± 8	170 ± 9	170 ± 10	0.890
Weight	72 ± 12	71 ± 12	78 ± 14	76 ± 19	0.428
BMI	24.7 ± 3.5	24.2 ± 3.4	27.0 ± 3.3	26.2 ± 5.8	0.109
SJ (66)	0.1 ± 0.5 ^(PsA)^	0.7 ± 1.6	0.9 ± 1.2 ^(PSO,HC)^	0.0 ± 0.0 ^(PsA)^	**<0.001**
TJ (68)	0.2 ± 0.4 ^(RA,PsA)^	2.1 ± 2.2 ^(HC)^	4.7 ± 7.9 ^(HC,PSO)^	0.4 ± 0.6 ^(PsA)^	**<0.001**
CRP	n/a	0.6 ± 0.5	0.4 ± 0.4	n/a	0.143
ESR	n/a	12 ± 8	19 ± 13	n/a	0.195
RF	n/a	14/19 (73.7)	n/a	n/a	
ACPA	n/a	15/19 (78.9)	n/a	n/a	
cDMARDs	n/a	14/18 (77.8)	9/17 (52.9)	3/16 (18.8)	**0.003**
bDMARDs	n/a	7/18 (38.9)	6/17 (35.3)	3/15 (20.0)	0.480
DAS28	n/a	1.53 ± 1.30	n/a	n/a	n/a
DAPSA	n/a	n/a	14.7 ± 11.4	n/a	n/a
MASES	0.2 ± 0.5	1.0 ± 1.6	0.8 ± 1.5	0.2 ± 0.8	0.096
PASI	n/a	n/a	3.5 ± 6.1	4.5 ± 6.8	0.636
NAPSI	n/a	n/a	2.6 ± 4.7	3.4 ± 9.5	0.701

Data are shown as mean ± SD or number (%). P value is the results of Kruskal-Wallis test. In apex brackets the groups with significant differences at *post-hoc* pairwise analyses are shown. Statistically significant p-values are shown in bold

### 3.1 Ultrasound and nailfold videocapillaroscopy assessment

Significant differences were detected in US and NVC parameters among the groups. Specifically, the groups exhibited distinct variations in total GS BUNES (p=0.002), plate GS BUNES (p<0.001), bed GS BUNES (p=0.026) and Wortsman classification (p<0.001). Pairwise analysis revealed lower GS BUNES, plate GS BUNES and Wortsman classification in the HC group compared to all other groups. Bed GS BUNES was lower in the HC group as compared to PSO group, with a similar trend observed for the other groups. No significant differences were observed among the groups concerning matrix GS BUNES, enthesitis score, PD BUNES and plate, matrix and bed thickness.

In terms of the NVC data, a significantly higher number of tortuous capillaries was observed in the PsA group (p=0.001). Pairwise analysis revealed a significant difference compared to the HC and PSO groups but not the RA group. Additionally, trends were noted for lower capillary density and a higher number of dilated capillaries in the PsA group (**Table 2**). Samples of NVC findings are shown in **Supplementary Figure S1**.

**Table 2:** Ultrasound and videocapillaroscopy parameters among groups.

	HC N=19	RA N=19	PsA N=18	PSO N=16	p
BUNES GS	0.36 ± 0.34 ^(RA,PsA,PSO)^	0.82 ± 0.59 ^(HC)^	0.78 ± 0.52 ^(HC)^	1.0 ± 0.62 ^(HC)^	**0.002**
BUNES GS Matrix	0.5 ± 0.6	1.1 ± 1.0	0.7 ± 0.9	1.4 ± 1.4	0.100
BUNES GS Plate	1.0 ± 0.8 ^(RA,PsA,PSO)^	2.6 ± 1.5 ^(HC)^	2.5 ± 1.2 ^(HC)^	2.9 ± 1.2 ^(HC)^	**<0.0001**
BUNES GS Bed	0.2 ± 0.3 ^(PSO)^	0.4 ±- 0.9	0.9 ± 1.3	0.9 ± 0.8 ^(HC)^	**0.026**
Wortsman	0.4 ± 0.4 ^(RA,PsA,PSO)^	1.2 ± 0.7 ^(HC)^	1.2 ± 0.8 ^(HC)^	1.3 ± 0.8 ^(HC)^	**<0.0001**
BUNES PD	1.4 ± 0.7	1.7 ± 0.6	2.1 ± 0.9	1.9 ± 1.1	0.091
BUNES PD Matrix	2.9 ± 1.9	3.7 ± 1.5	4.3 ± 1.6	3.9 ± 2.1	0.058
BUNES PD Bed	4.2 ± 2.3	5.0 ± 1.9	6.0 ± 2.9	5.7 ± 3.4	0.171
Plate thickness	0.56 ± 0.04	0.56 ± 0.06	0.58 ± 0.15	0.58 ± 0.06	0.161
Matrix thickness	2.92 ± 0.32	2.91 ± 0.49	3.13 ± 0.53	2.96 ± 0.35	0.621
Bed thickness	1.68 ± 0.27	1.71 ± 0.28	1.77 ± 0.39	1.69 ± 0.21	0.931
Enthesis GS	0.7 ± 0.6	0.7 ± 0.5	1.0 ± 0.6	0.8 ± 0.4	0.243
Capillary density	8.1 ± 0.8	8.4 ± 1.3	7.7 ± 0.6	8.5 ± 1.5	0.099
Microhaemorrages	0.6 ± 0.8	0.3 ± 0.6	0.5 ± 0.7	0.2 ± 0.3	0.425
Tortuosities	0.3 ± 0.6 ^(PsA)^	0.8 ± 1.1	1.6 ± 1.2 ^(PSO)^	0.6 ± 0.9 ^(PsA)^	**0.001**
Ectasia	1.9 ± 0.4	1.9 ± 0.5	2.4 ± 0.8	1.9 ± 0.5	0.059
Ramifications	0.03 ± 0.12	0.10 ± 0.35	0.07 ± 0.12	0.05 ± 0.08	0.247

Data are shown as mean ± SD or number (%). P value is the results of Kruskal-Wallis test. In apex brackets the groups with significant differences at *post-hoc* pairwise analyses are shown. Statistically significant p-values are shown in bold.

### 3.2 Inter-rater reliability

Inter-rater reliability of semiquantitative US scores was assessed and yielded varying results. Reliability was classified as moderate for GS BUNES of the nail plate, substantial for Wortsman type and GS enthesitis score, almost perfect for PD BUNES of the bed and matrix, and perfect for GS BUNES of the nail bed and matrix (**Table 3**).

**Table 3:** Inter-rater reliability of US parameters.

Parameter	Cohen’s kappa	p
Wortsman classification	0.633	<0.001
Matrix GS BUNES	1.0	<0.001
Plate GS BUNES	0.600	0.006
Bed GS BUNES	1.0	<0.001
Matrix PD BUNES	0.857	<0.001
Bed PD BUNES	0.835	<0.001
Enthesitis GS	0.620	<0.001

### 3.3 Clustering model and factor analysis

The number of clusters, according to the elbow method, was set at 5 (**Supplementary Figure S2**). The results of the preliminary k-means and DEC models are shown in **Supplementary Figure S3**. The silhouette score at five clusters for the k-means and DEC clustering models was 0.27 and 0.17, respectively. The following analyses were therefore carried out employing a k-means model.

**Figure 2:**
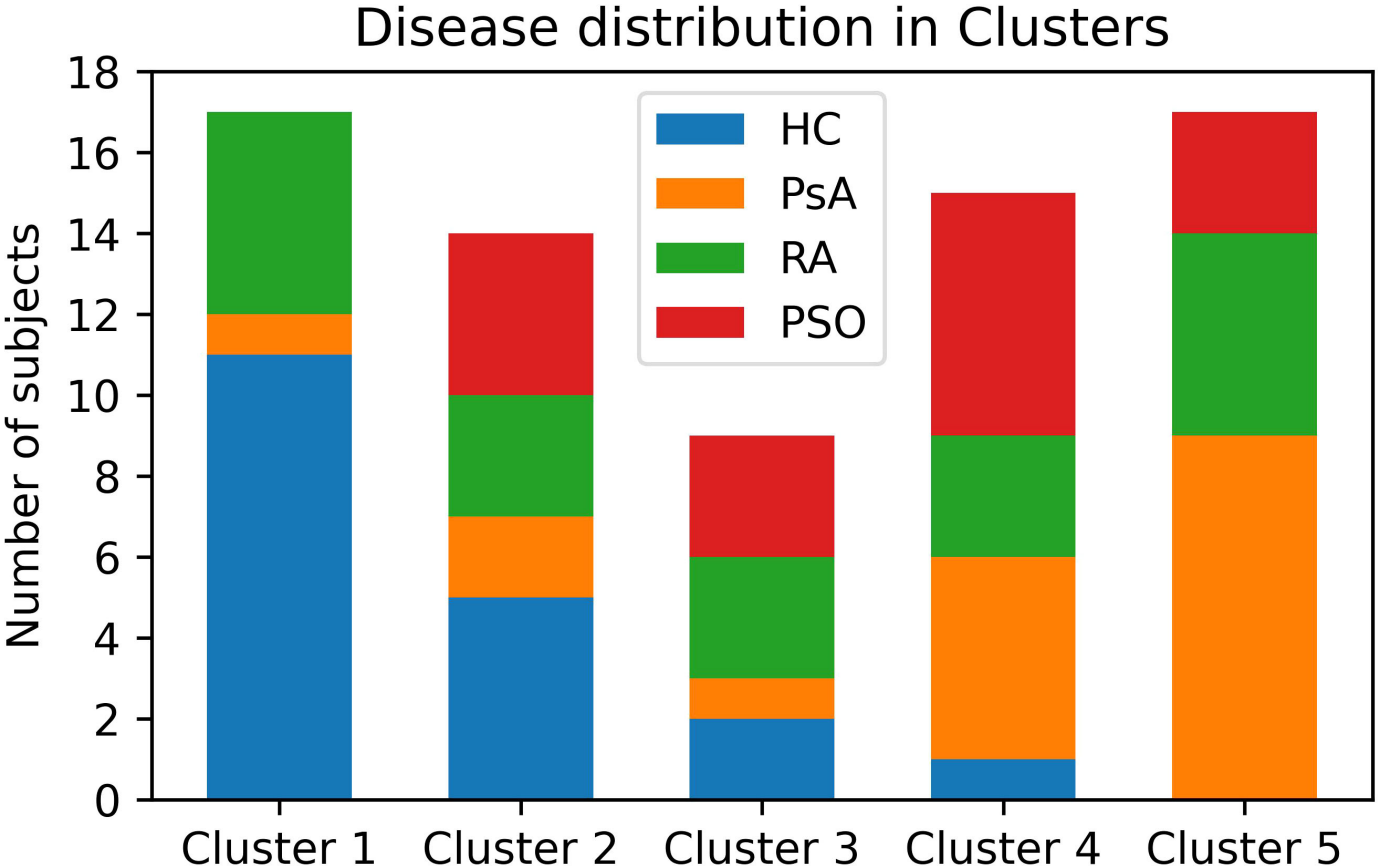
Distribution of patients and HC among the clusters. PsA patients are mostly clustered in Clusters 4 and 5. HC are mostly clustered in Clusters 1 and 2. PSO subject clustered similarly to PsA, though with a less marked preference for Clusters 4 and 5. RA patients are almost evenly distributed among all clusters. HC, healthy controls; PsA, psoriatic arthritis; PSO, skin psoriasis; RA, rheumatoid arthritis.

The clustering model effectively segregated the subjects into five distinct clusters (C1 to C5). Cluster 1 (C1) included 11 HC, 1 PsA, and 5 RA subjects. C2 included 5 HC, 2 PsA, 3 RA, and 4 PSO subjects. C3 included 2 HC, 1 PsA, 3 RA, and 3 PSO. C4 included 1 HC, 5 PsA, 3 RA, and 6 PSO subjects. C5 included 9 PsA, 5 RA, and 3 PSO patients (**Figure 2**).

To further understand how the variables collected by US and NVC influenced the clustering model we tested the data with Bartlett’s sphericity test and KMO. The Chi-squared statistic of Bartlett’s sphericity test was 1058.47 (p = 1.37 x 10^-142^). KMO test result was 0.53, which was considered just adequate for factor analysis (21).

**Figure 3:**
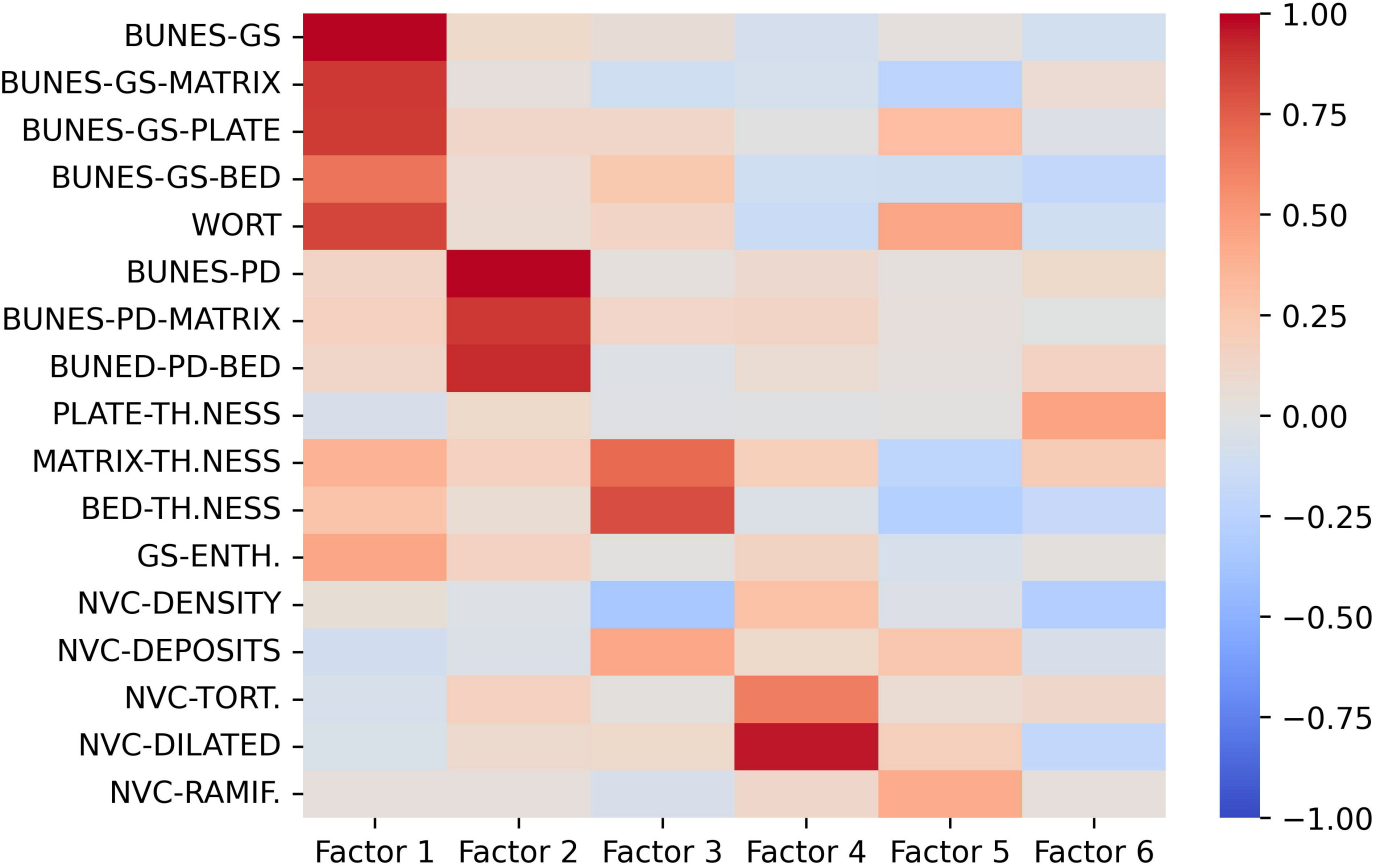
Loading values of US and NVC variables on the first 6 factors. Gray-scale US parameters mostly load on Factor 1, US PD features mostly load on Factor 2, Nail-enthesis quantitative measures mostly load on Factor 3, NVC-detected ectasia and tortuosities mostly load on Factor 4. The remaining variables do not seem to provide major contribution to the clustering model. BUNES, Brown University Nail Enthesis Scale; GS, grayscale; PD, Power-Doppler.

Factor eigenvalues were determined and six factors were identified based on the defined criteria (**Supplementary Figure S4**). Semiquantitative gray-scale US parameters (BUNES scores and Wortsman type) loaded on factor 1, PD BUNES scores loaded on factor 2. Matrix and bed thickness loaded on factor 3 and the number of tortuous and dilated capillaries at NVC loaded on factor 4. Loadings of factor 5 and 6 and of the remaining variables were below 0.5 (**Figure 3**). For further clarity, Factor 1 and 2 loadings were plotted and are shown in **Supplementary Figure S5**. These factors elucidated the interplay between various US and NVC parameters, providing crucial insights into the overall distribution of the subjects among the distinct clusters (**Figure 3**).

## 4 Discussion

This pilot study aimed to investigate the distinctive features of the nail-enthesis complex among PsA, PSO, RA, and HC groups. The study focused on the structural and vascular alterations observed through the combined use of US and NVC. Notably, the study yielded several important observations that contribute to the understanding of these conditions.

The assessment of structural changes within the nail-enthesis complex, specifically through the implementation of the BUNES score and Wortsman classification, revealed significant alterations primarily in the three patient groups. However, intriguingly, no substantial differences were detected in terms of plate, bed, and matrix thickness among the groups. Previous studies have reported different findings regarding the thickness of these structures, with conflicting results (3, 22–25). To the best of our knowledge, no study employed the GS BUNES score, thus preventing any comparison with our results (26, 27).

Surely, the assessment of very minute structural changes may limit their reliable detection and findings may be influenced by cohort-specific variations. Moreover, the influence of nail involvement by PSO underscores the complexity of utilizing plate thickness as a differentiating factor. In fact, its value may depend on the prevalence and severity of nail involvement in the investigation cohort. In our case, for instance, nail involvement was overall absent or very mild. The employment of a semiquantitative score, such as BUNES, emerges as a more feasible and practical approach for clinical applications.

Despite the absence of significant differences in nail unit vascularization, trends in line with existing literature were observed, with higher degrees of vascularization noted in PsA and PSO, albeit without statistical significance (24, 27, 28). These trends align with the prevailing understanding of altered neoangiogenesis and inflammatory damage to capillaries in PsA and PSO.

Similarly, a trend toward higher enthesitis scores in the PsA group is in line with previous findings, though not without controversies (5, 29).

Notably, the interplay between the nail unit and the distal enthesis of the extensor digitorum tendon is well-documented, emphasizing the association between the severity of nail involvement and the presence of enthesitis (29–31), along with a correlation between systemic enthesitis and US nail changes (11).

Similarly to what was described by other Authors, we found a significantly increased number of tortuous capillaries in PsA, which were mostly short and “bushy” (7, 32). Although not statistically significant, our results on capillary density and ectasia are in line with the available literature (32, 33). Nail fold vascular modifications in PsA are believed to be the results of altered neoangiogenesis and inflammatory damage to capillaries which is also a process taking place in RA, although to a lesser extent, and may explain why the study failed to find differences between PsA and RA (34, 35). Additionally, NVC changes in RA have already been described (7). Interestingly, the results of our study are also in line with the findings by Fukasawa et al. (36) who observed a higher rate of development of musculoskeletal involvement in PSO patients who displayed microhaemorrhages and capillary ectasia at the nailfold. Although not statistically significant due to the Bonferroni correction for multiple comparisons, our PsA cohort shows higher values for both variables compared to PSO patients. Similarly, we also found a significantly higher prevalence of tortuous capillaries in PsA than PSO, which was not assessed by Fukasawa et al. but may represent another accompanying feature pointing in the same direction.

The utilization of a clustering algorithm provided valuable insights into the distinct patterns observed among the different groups. The clustering model effectively distinguished HC subjects from PsA patients, further suggesting potential overlap between PSO and PsA groups, indicating a continuum between the two conditions. Nonetheless, a significant proportion of PSO patients were included in C2, suggesting an intermediate phenotype between PsA and HC, in line with previous data (3, 5, 27, 29).

The results of the clustering algorithm with regards to RA patients were somewhat surprising. Considering the general concept that RA does not involve DIJ due to the absence of a synovial lining, findings similar to those of HC were expected. Although the presence of vascular changes has been previously described and is supported by well-known disruption of neoangiogenesis, nail-enthesis complex US features were, to a certain extent, very similar to those of PsA and PSO. The distribution pattern of RA patients revealed subsets of patients with characteristics similar to both HC and PsA/PSO groups. This intricate pattern highlights the complexities underlying the distinct manifestations of these conditions and additional studies aimed at exploring differences among these subsets are warranted.

The use of DEC clustering algorithm did not provide any additional value to the analysis, likely due to the low dimensionality of the dataset.

Factor analysis provided further insights into the interrelationship between the ultrasound and NVC parameters, emphasizing the collinearity observed in the US-detected characteristics of the nail-enthesis complex as all gray-scale parameters and PD-associated parameters loaded on factor 1 and 2, respectively. The thickness of the bed and matrix had a significant impact on the model. However, as clearly shown in **Table 2** and even more clearly in **Supplementary Table S1**, the assessment of the bed and matrix thickness underscored the limited discriminative power of these parameters, due to their minimal mean differences and susceptibility to measurement errors. Additionally, the time required to acquire such parameters makes them hardly applicable. Finally, tortuous and dilated capillaries also significantly contributed to the clustering model.

In summary, the results of the study highlighted notable distinctions in the nail US and NVC assessments among the PsA, PSO, RA, and HC groups. The clustering model further underscored the potential of the combined ultrasound and NVC data to effectively discern specific patterns and characteristics within the study population. In fact, the clustering algorithm based on the combined US and NVC evaluation found marked differences between PsA and HC, indicating clear distinctions between these groups. Additionally, the PSO group showed similarities to PsA, suggesting a continuum or overlap between these conditions. However, surprisingly, the combined evaluation was not able to effectively differentiate RA from the other conditions, highlighting the complexities and unexpected potential overlaps in terms of nail-enthesis complex changes between RA and psoriatic disease.

The study’s strengths include the rigorous enrolment process, the strict blinding procedures, and the innovative integration of two distinct diagnostic methods. Additionally, the use of a semiquantitative US score greatly improves the feasibility of the assessment compared to quantitative measures demonstrating high inter-rater reliability. However, some limitations, such as the limited cohort size due to its pilot nature and the predominantly low disease activity of the participants – limiting the potential impact of disease activity on the variables measured – should be acknowledged. For the same reasons, reproducibility of the clustering outcome may be questioned; however, the purpose of the clustering model was to investigate the role of US and NVC parameters and their mutual relationship in an unsupervised model, with the prospect of detecting variable potentially applicable for routine use. Future research endeavors should aim to explore the identified variables in prospective trials, thereby potentially predicting the evolution of undifferentiated early arthritis and the onset of arthritis in PSO patients.

## Data Availability

The raw data supporting the conclusions of this article will be made available by the authors, without undue reservation.
