# Supplementary material for: Combined semiquantitative nail-enthesis complex ultrasonography and capillaroscopy in psoriasis and psoriatic arthritis
Source: Front Immunol. 2025 Jan 14;15:1505322. doi: 10.3389/fimmu.2024.1505322 (PMC11772180; doi:10.3389/fimmu.2024.1505322)

**Supplementary Material**

**Supplementary Table S1. Characteristics of the clusters**

|  | **Cluster 1**  **N=17** | **Cluster 2**  **N=14** | **Cluster 3**  **N=9** | **Cluster 4**  **N=15** | **Cluster 5**  **N=17** | **P** |
| --- | --- | --- | --- | --- | --- | --- |
| Age | 53 ± 12 | 58 ± 12 | 57 ± 13 | 59 ± 9 | 60 ± 11 | 0.365 |
| Sex (M) | 5/17 (29.4) | 6/14 (42.9) | 3/9 (33.3) | 9/15 (60.0) | 12/17 (70.6) | 0.107 |
| Disease duration | 7.6 ± 5.4 | 18.6 ± 16.8 | 8.6 ± 6.7 | 6.7 ± 7.1 | 11 ± 12 | 0.302 |
| Height | 169 ± 8 | 168 ± 9 | 170 ± 6 | 173 ± 11 | 172 ± 8 | 0.416 |
| Weight | 67 ± 11 | 72 ± 15 | 71 ± 12 | 79 ± 16 | 80 ± 14 | **0.05** |
| BMI | 23.5 ± 3.7 | 25.6 ± 5.0 | 24.5 ± 3.5 | 26.4 ± 4.5 | 26.9 ± 3.4 | 0.089 |
| SJ (66) | 0.9 ± 1.8 | 0.1 ± 0.4 | 0.1 ± 0.3 | 0.6 ± 1.2 | 0.3 ± 0.6 | 0.754 |
| TJ (68) | 1.3 ± 2.3 | 0.4 ± 0.6 | 0.4 ± 0.7 | 4.5 ± 8.7 | 2.1 ± 2.4 | 0.084 |
| CRP | 0.3 ± 0.2 | 0.3 ± 0.2 | 0.5 ± 0.6 | 0.5 ± 0.3 | 0.7 ± 0.5 | 0.233 |
| ESR | 9 ± 6 | 15 ± 11 | 13 ± 9 | 12 ± 9 | 19 ± 13 | 0.474 |
| RF | 3/5 (60.0) | 3/3 (100) | 2/3 (66.7) | 2/3 (66.7) | 4/5 (80.0) | 0.771 |
| ACPA | 5/5 (100) | 3/3 (100) | 3/3 (100) | 1/3 (33.3) | 3/5 (60.0) | 0.100 |
| cDMARDs | 5/5 (100) | 5/9 (55.6) | 2/4 (33.3) | 5/14 (35.7) | 9/17 (52.9) | 0.138 |
| bDMARDs | 1/5 (20.0) | 3/9 (33.3) | 2/5 (40.0) | 3/14 (33.3) | 7/17 (41.2) | 0.761 |
| MASES | 0.7 ± 1.5 | 0.6 ± 1.1 | 0.2 ± 0.7 | 0.7 ± 1.4 | 0.6 ± 1.2 | 0.915 |
| BUNES GS | 0.2 ± 0.2^(3,4,5)^ | 0.5 ± 0.2^(4)^ | 0.9 ± 0.3^(1)^ | 1.6 ± 0.4^(1,2,5)^ | 0.6 ± 0.3^(1,4)^ | **<0.0001** |
| BUNES GS Matrix | 0.3 ± 0.4^(4)^ | 0.5 ± 0.5^(4)^ | 1.1 ± 0.8 | 2.4 ± 0.9^(1,2,5)^ | 0.5 ± 0.5^(4)^ | **<0.0001** |
| BUNES GS Plate | 0.6 ± 0.5^(3,4,5)^ | 1.8 ± 0.8^(4)^ | 2.9 ± 1.0^(1)^ | 4.0 ± 0.7^(1,2,5)^ | 2.2 ± 0.9^(1,4)^ | **<0.0001** |
| BUNES GS Bed | 0.1 ± 0.5^(4)^ | 0.2 ± 0.4^(4)^ | 0.4 ± 0.4^(4)^ | 1.9 ± 1.2^(1,2,3)^ | 0.4 ± 0.6 | **<0.0001** |
| Wortsman | 0.3 ± 0.2^(3,4,5)^ | 0.8 ± 0.5^(4)^ | 1.3 ± 0.6^(1)^ | 2.0 ± 0.7^(1,2,5)^ | 1.0 ± 0.5^(1,4)^ | **<0.0001** |
| BUNES PD | 1.2 ± 0.5^(2,4,5)^ | 2.3 ± 0.5^(1,3)^ | 0.6 ± 0.6^(2,4,5)^ | 2.3 ± 0.9^(1,3)^ | 2.0 ± 0.4^(1,3)^ | **<0.0001** |
| BUNES PD Matrix | 2.4 ± 1.0^(2,4,5)^ | 4.6 ± 1.0^(1,3)^ | 1.1 ± 1.4^(2,4,5)^ | 5.0 ±1.7^(1,3)^ | 4.4 ± 0.9^(1,3)^ | **<0.0001** |
| BUNES PD Bed | 3.6 ± 1.7^(2,4,5)^ | 6.8 ± 2.0^(1,3)^ | 1.8 ± 1.7^(2,4,5)^ | 6.8 ± 3.0^(1,3)^ | 5.9 ± 1.5^(1,3)^ | **<0.0001** |
| Plate thickness | 0.54 ± 0.04^(2)^ | 0.65 ± 0.15^(1,5)^ | 0.57 ± 0.04 | 0.56 ± 0.06 | 0.55 ± 0.05^(2)^ | **0.001** |
| Matrix thickness | 2.72 ± 0.38^(4)^ | 3.03 ±0.36 | 2.68 ± 0.25^(4)^ | 3.36 ± 0.45^(3,1)^ | 3.02 ± 0.34 | **<0.0001** |
| Bed thickness | 1.60 ± 0.23 | 1.72 ± 0.28 | 1.55 ± 0.16 | 1.90 ± 0.38 | 1.72 ± 0.24 | **0.046** |
| Enthesis GS | 0.4 ± 0.3^(4,5)^ | 0.8 ± 0.6 | 0.7 ± 0.3 | 1.2 ± 0.6^(1)^ | 1.0 ± 0.5^(1)^ | **0.001** |
| Capillary density | 7.9 ± 0.9 | 7.6 ± 1.0 | 8.6 ± 0.6 | 8.0 ± 1.1 | 8.7 ± 1.3 | **0.041** |
| Microhaemorrages | 0.3 ± 0.7 | 0.5 ± 0.8 | 0.3 ± 0.3 | 0.4 ± 0.7 | 0.5 ± 0.7 | 0.842 |
| Tortuosities | 0.5 ± 0.8^(5)^ | 0.2 ± 0.5^(5)^ | 0.3 ± 0.5^(5)^ | 0.7 ± 0.9^(5)^ | 1.9 ± 1.2^(1,2,3,4)^ | **<0.0001** |
| Ectasia | 1.8 ± 0.5^(5)^ | 1.6 ± 0.3^(5)^ | 1.9 ± 0.4 | 1.9 ± 0.5^(5)^ | 2.6 ± 0.6^(1,2,4)^ | **<0.0001** |
| Ramifications | 0.04 ± 0.12 | 0.03 ± 0.05 | 0.22 ± 0.50 | 0.04 ± 0.10 | 0.06 ± 0.09 | 0.515 |

**Supplementary Figure S2.** Sample NVC pictures. **(A)** Normal NVC pattern with “hairpin” shaped capillaries, ≤ 2 crossings and normal density. **(B)** NVC pattern displaying the most significant change found in PsA patients, i.e. tortuous capillary with 3 crossings between arterial and venous branches (solid black arrow) or multiple crossings producing a “bushy” aspect (void black arrow).


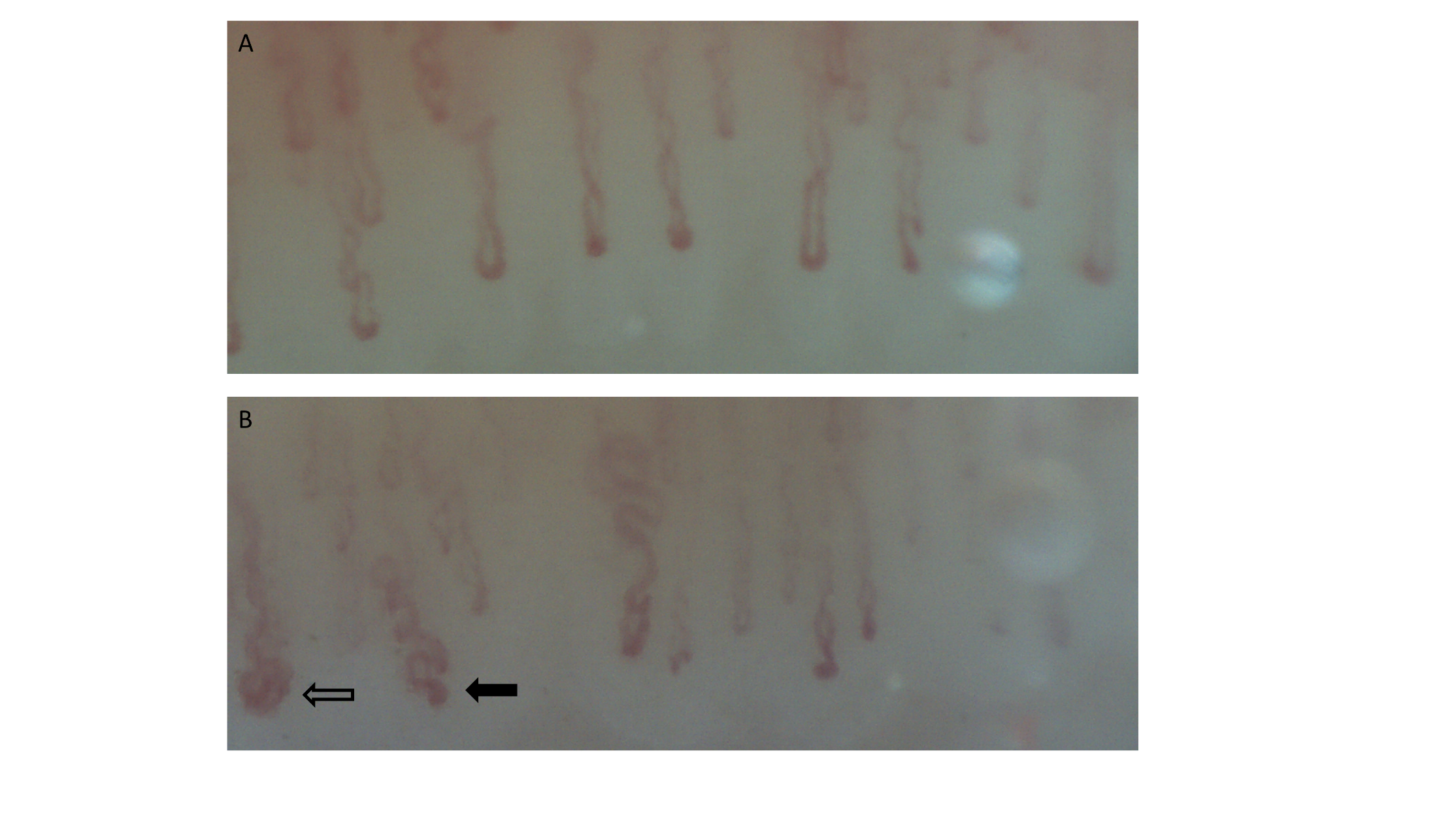


**Supplementary Figure S3. Definition of the number of clusters according to the elbow method.** The plot shows the sum of squared errors against the number of clusters. The number of clusters was set at 5. *SSE, Sum of Squared Errors*

**
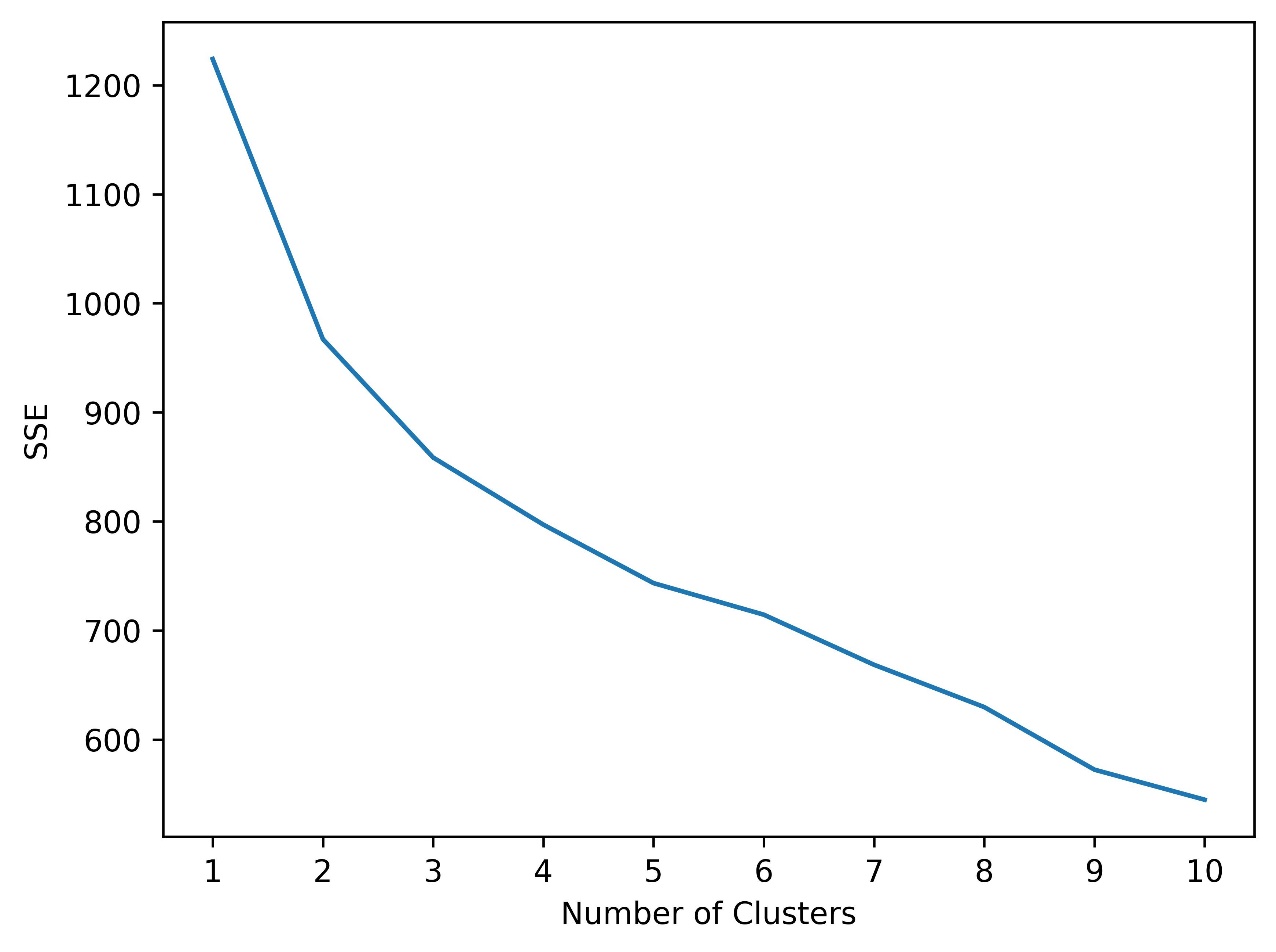
**

**Supplementary Figure S4.** T-SNE plot of k-means model at 5 clusters; silhouette score 0.27 (A). T-SNE plot of DEC model at 5 clusters; silhouette score 0.17 (B)

**
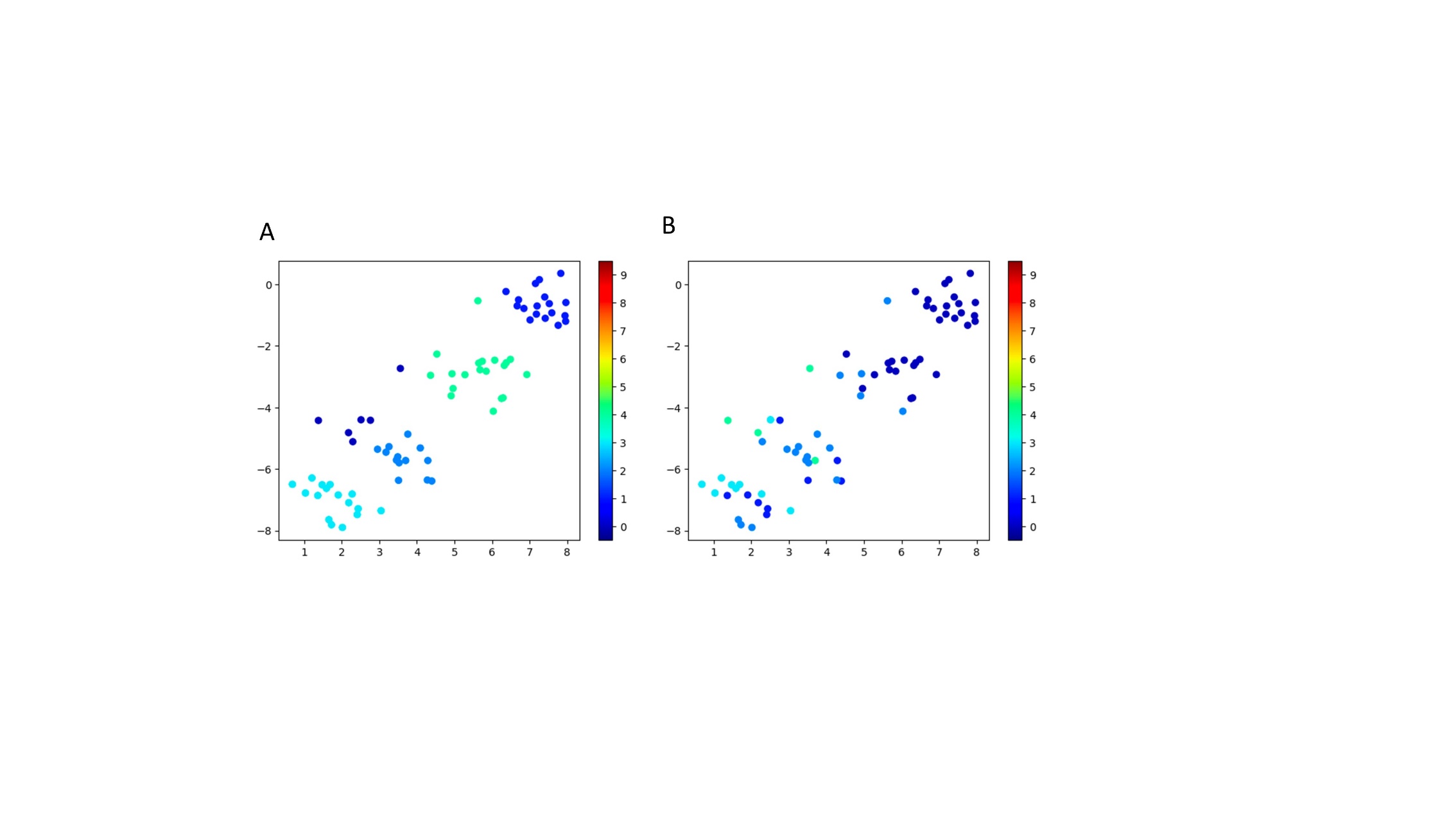
**

**Supplementary Figure S5. Scree plot.** Eigenvalue of each factor is shown on the y axis. Factors with eigenvalue ≥ 1 were further investigated.

**
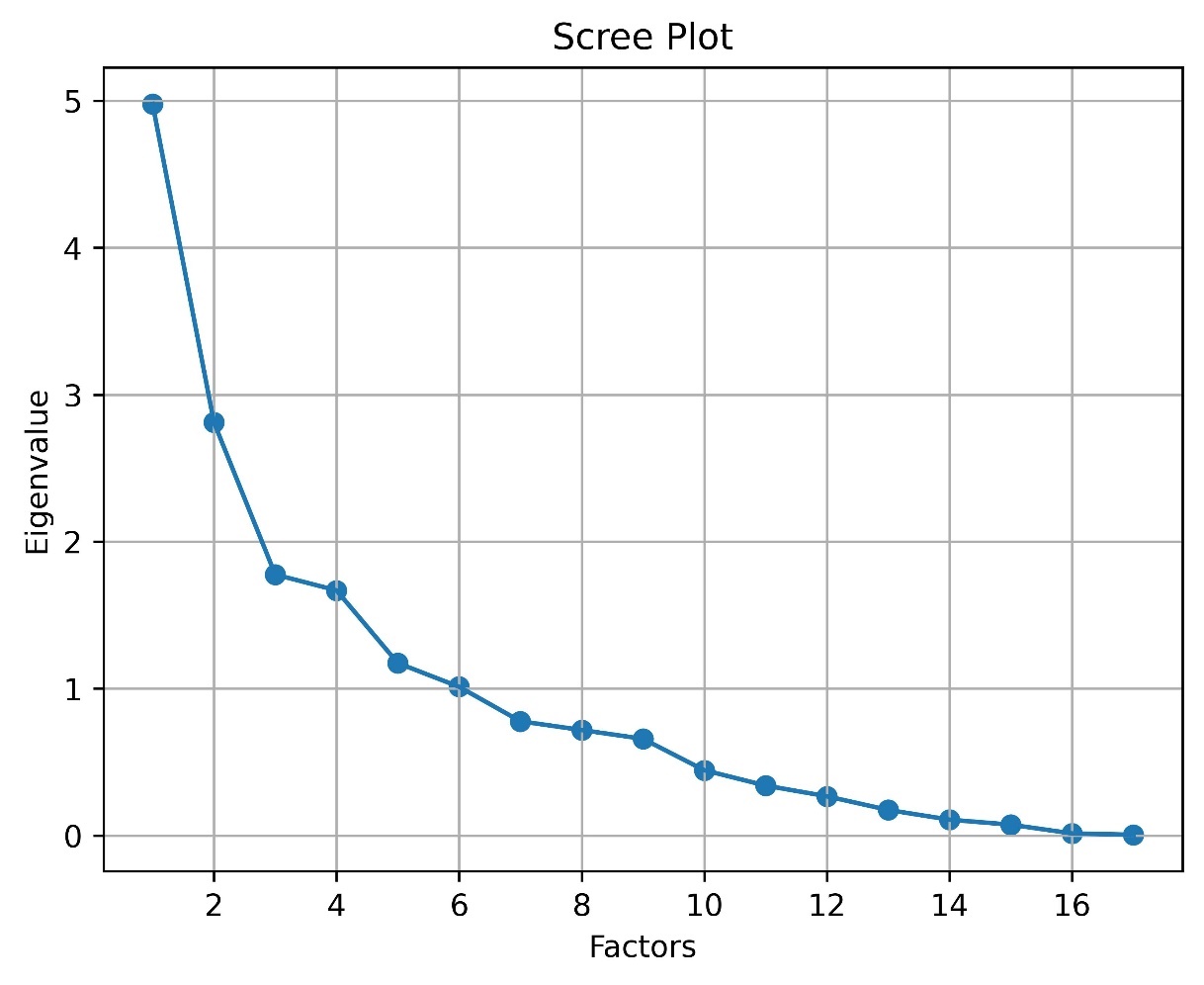
**

**Supplementary Figure S6. Loadings of factors 1 and 2.** The plot shows the loadings of the variables for the first two factors. The yellow area represents loadings ≥ 0.6


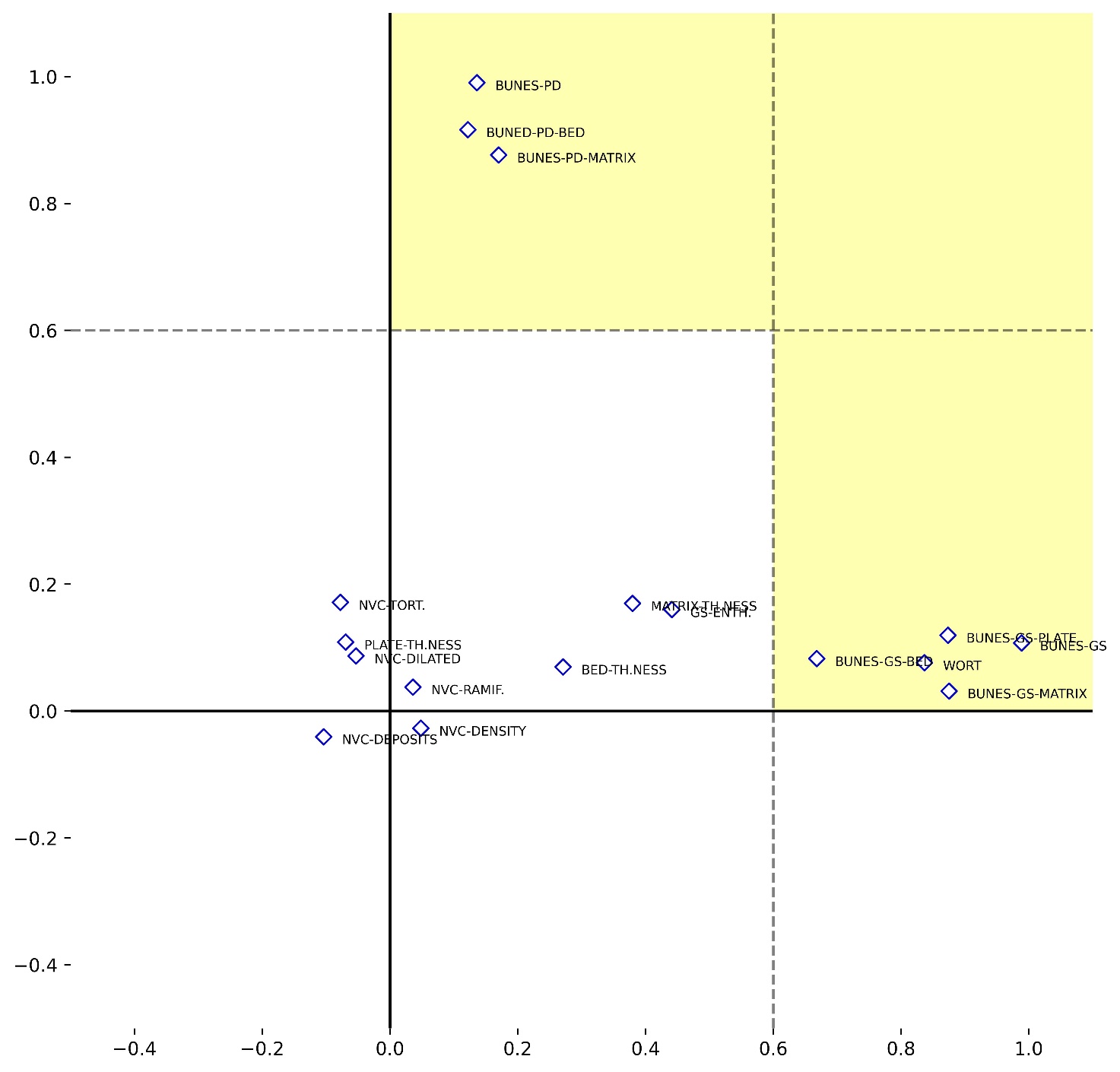

Supplement: Supplementary file 1 [file DataSheet1.docx]
